# Land-Use Change and Management Intensification Is Associated with Shifts in Composition of Soil Microbial Communities and Their Functional Diversity in Coffee Agroecosystems

**DOI:** 10.3390/microorganisms10091763

**Published:** 2022-08-31

**Authors:** Karen Carrasco-Espinosa, Morena Avitia, Alberto Barrón-Sandoval, Thalita F. Abbruzzini, Ulises Isaac Salazar Cabrera, Denise Arroyo-Lambaer, Adriana Uscanga, Julio Campo, Mariana Benítez, Ana Wegier, Julieta A. Rosell, Frédérique Reverchon, Gerardo Hernández, Karina Boege, Ana E. Escalante

**Affiliations:** 1 Laboratorio Nacional de Ciencias de la Sostenibilidad, Instituto de Ecología, Universidad Nacional Autónoma de México, Mexico City 04510, Mexico; 2 Programa de Doctorado en Ciencias Biomédicas, Universidad Nacional Autónoma de México, Mexico City 04510, Mexico; 3 Department of Ecology and Evolutionary Biology, University of California, Irvine, CA 92697, USA; 4 Laboratorio de Biogeoquímica Terrestre y Clima, Instituto de Ecología, Universidad Nacional Autónoma de México, Mexico City 04510, Mexico; 5 Laboratorio de Restauración Ecológica, Instituto de Biología, Universidad Nacional Autónoma de México, Mexico City 04510, Mexico; 6 Laboratorio de Genética de la Conservación, Jardín Botánico, Instituto de Biología, Universidad Nacional Autónoma de México, Mexico City 04510, Mexico; 7 Red de Estudios Moleculares Avanzados, Instituto de Ecología A.C., Pátzcuaro 91070, Mexico; 8 Centro Agroecológico del Café A.C. Clúster Biomimic-Inecol, Xalapa Enríquez Centro, Veracruz 91000, Mexico; 9 Departamento de Ecología Evolutiva, Instituto de Ecología, Universidad Nacional Autónoma de México, Mexico City 04510, Mexico

**Keywords:** land-use change, management intensity, soil microbial communities, carbon utilization

## Abstract

Despite the central role of microorganisms in soil fertility, little understanding exists regarding the impact of management practices and soil microbial diversity on soil processes. Strong correlations among soil microbial composition, management practices, and microbially mediated processes have been previously shown. However, limited integration of the different parameters has hindered our understanding of agroecosystem functioning. Multivariate analyses of these systems allow simultaneous evaluation of the parameters and can lead to hypotheses on the microbial groups involved in specific nutrient transformations. In the present study, using a multivariate approach, we investigated the effect of microbial composition (16SrDNA sequencing) and soil properties in carbon mineralization (C_MIN_) (BIOLOG™, Hayward, CA, USA) across different management categories on coffee agroecosystems in Mexico. Results showed that (i) changes in soil physicochemical variables were related to management, not to region, (ii) microbial composition was associated with changes in management intensity, (iii) specific bacterial groups were associated with different management categories, and (iv) there was a broader utilization range of carbon sources in non-managed plots. The identification of specific bacterial groups, management practices, and soil parameters, and their correlation with the utilization range of carbon sources, presents the possibility to experimentally test hypotheses on the interplay of all these components and further our understanding of agroecosystem functioning and sustainable management.

## 1. Introduction

Soil is one of the most diverse and complex environmental matrices on the planet [1]. The impact of soil microbial communities on ecosystem functions, such as nutrient cycling, has long been recognized [2,3,4]. The relationship between microbial communities and ecosystem functioning is of particular interest in agricultural ecosystems, which represent 34% of the world’s ice-free land surface [5]. One of the main concerns regarding the functioning of managed ecosystems is the impact of different human intensification practices that include mechanized operations such as tillage, irrigation, and the increasing use of fertilizers and pesticides [6,7,8]. Recent studies have investigated the effects of intensification practices on the diversity and composition of soil microbial communities and the subsequent potential impacts on nutrient transformations [9,10,11,12,13]. However, most reports on management intensification practices have focused on annual cropping systems, and little is known about the impact of management intensification on soil microbial communities in perennial agroforestry systems, despite the importance of understanding these relationships for their sustainable management [14,15].

Even though the relevance of microorganisms to nutrient transformations is widely recognized, traditional approaches associated with the culture isolation of specific groups or species have hindered progress in the understanding of the relationship between changes in microbial diversity and its functional consequences [16,17,18]. In contrast, culture-independent techniques, such as 16SrDNA amplicon sequencing and shotgun metagenomics, can provide coarse information of the diversity and composition of entire microbial communities in the context of environmental conditions and management practices, which has advanced our knowledge in the field [19,20,21]. In addition, coupling culture-independent approaches with the direct measurement of specific community-level functions, along with multivariate statistical analyses, can lead to hypotheses to be further tested in the search for a mechanistic understanding of the relationship of diversity and function [22,23].

Shifts in soil microbial biomass and composition have been related to environmental parameters, land use, and management practices. A broad range of soil properties such as depth, water retention [24], salinity [25], texture [26], pH [27,28], and vegetation composition [29] have been studied as predictors of microbial community composition and diversity [30]. Previous studies have shown strong correlations among changes in soil microbial communities, land use, or management practices and microbially mediated ecosystem functions, i.e., N fixation, organic matter decomposition, and soil organic carbon mineralization (C_MIN_) [4,31,32,33,34,35,36,37]. For instance, it has been shown that, under conventional management (characterized by the use of synthetic fertilizers and pesticides), mineralization rates are affected, increasing the risk of mineral nitrogen loss and reduced carbon soil [38]. It has long been debated if microbially mediated ecosystem functions can be attributed to the presence of specific microbial groups or if composition matters in terms of function at the scale of communities [19,34,39,40,41]. In this regard, it is key to consider microbial diversity not only from the taxonomic perspective but in the context of its functional and ecological roles [42,43]. For instance, conversions of forests to agricultural systems, associated with lower soil pH and higher soil C:N ratio, lead to increases in copiotrophic compared to oligotrophic microbial groups (i.e., Proteobacteria vs. Acidobacteria, Bacteroidetes vs. Actinobacteria) [44].

The present study aimed to investigate the impact of changes in land use and the intensity of soil management practices on the presence and abundance of bacterial groups in soil communities and their relationship with the degradation of specific carbon sources as a measure of function and a proxy for C_MIN_. We hypothesize that, in the context of land-use change and intensification practices, shifts in the microbial composition of soil communities will be observed, coupled with a reduction in their functional diversity (C source range) in terms of carbon source degradation capacity. For this, we used a multivariate analysis approach to evaluate the importance of soil properties, management characteristics, and composition, and the functional diversity of microbial communities on C_MIN_. Specifically, we followed a culture-independent approach and sequencing of 16S rDNA and looked at C_MIN_ for different C sources (BIOLOG GEN-III), as a direct measure of community-level functions, in coffee agricultural ecosystems along a gradient of management in Central Veracruz, Mexico. Through this approach, we were able to identify correlations between microbial community composition, soil environmental variables and management categories, and functional outcomes. We found microbial composition shifts associated with land-use change and specific soil parameters, along with a reduction in the C source utilization range as management practices intensified.

## 2. Materials and Methods

### 2.1. Study Sites

To evaluate the relationship between coffee management practices, soil properties, microbial diversity, and their impact on soil C_MIN_, we sampled soil from three localities with coffee agroecosystems varying in environmental, edaphic, and management characteristics. We also sampled sites with secondary vegetation as “non-managed” or “reference” sites for each locality (*n* = 12).

**Figure 1:**
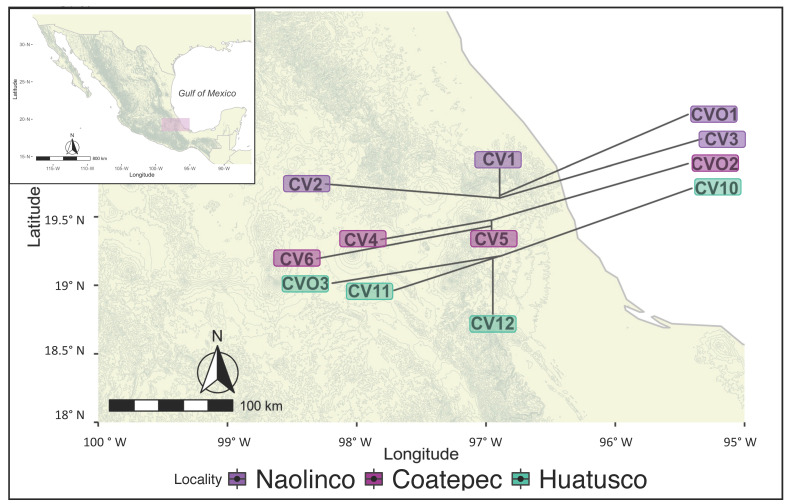
Sampling sites in Veracruz, Mexico. Managed coffee agroecosystems plots (CV) and non-managed plots (CVO) were sampled at three localities: Naolinco (CV1–3 and CVO1), Coatepec (CV4–6 and CVO2), and Huatusco (CV10–12 and CO3) in Veracruz, Mexico.

The study area included three localities in the mountainous region of central Veracruz, Mexico, ranked second in coffee production in the country [45]. The localities were Coatepec (19°47′ N, 96°96′ W), Huatusco (19°21′ N, 96°94′ W), and Naolinco (19°64′ N, 96°89′ W) (Figure 1), and within each locality, each site was considered a replicate. Long-term climatic data from weather stations across the state of Veracruz indicate that the entire region is characterized by a distinct period of low precipitation [46]. The climate in the region is warm and subhumid and supports tropical montane wet forests and tropical rainforests.

The average annual precipitation occurs mostly during the rainy season (June–November) and it changes across localities (Naolinco 1690 ± 112 mm; Coatepec 1755 ± 98 mm; Huatusco 2020 ± 135 mm). Mean temperature across localities is 19.3 ± 2.4 °C in Coatepec, 18.5 ± 2.2 °C in Huatusco, and 17.1 ± 2.1 °C in Naolinco, and altitude ranges from 1188 to 1142 m. Soils are Andosols: SilAndic [47].

To qualify the intensity of coffee management practices, we constructed a management intensity index based on previous characterizations of practices in coffee plantations [48,49,50,51,52]. This index considers nutrient inputs (chemical fertilizers), mechanization practices (tractor, yoke and irrigation), and pest/weed control strategies (pesticides, herbicides, and fungicides). The information needed to calculate the index was obtained through semi-structured surveys of the coffee producers. Three levels of intensification were recognized (Table S1): none (0), low (0–0.25), medium (0.25–0.5), and high (0.5–1.0) (Equations (1) and (2)). Reference sites were assigned an index of zero (0.0).

(1) $$\mathrm{Management}\;\mathrm{Index}\left(x\right)=\overset{n}{\underset{i=1}{\sum }}x_{i}$$

Equation (1). Management Index (MI) of categorical management intensity based on practices and inputs, where *x_i_* is each categoric component of the survey, and *n* corresponds to the management variables.

(2) $$Management\;Category=\frac{MI_{max}-MI_{min}}{n}$$

Equation (2). Management categorization of coffee agricultural ecosystems, where *MI_max_* and *MI_min_* are the maximum and minimum average values obtained through surveys, and n is the number of management categories (low, medium, high).

### 2.2. Sampling

Sampling occurred during the rainy season (September 2016). At each site, a 30 × 30 m plot was established with three equidistant transects (10 m apart) from which three equidistant soil cores of 7.5 cm in diameter and 10 cm in depth were sampled. The three sampling points per transect were combined into one composite sample for a total of three samples per plot in each locality (*n* = 36). Samples were then divided into two subsamples, one for soil physicochemical characterization and the other for both microbial diversity analyses and C_MIN_ assays. Subsamples for soil characterization analyses were stored in black plastic bags and refrigerated until processed. Subsamples for microbial diversity and C_MIN_ assays were combined into one composite sample per plot (*n* = 12). Samples for microbial diversity analyses were preserved with RNA later^TM^ (Sigma, St. Louis, MO, USA) (1:1) in 50 mL centrifuge tubes; RNA later^TM^ was removed before storage at −80 °C until DNA extraction. Samples for C_MIN_ analyses were stored in 50 mL centrifuge tubes and kept at room temperature for two days before incubation in BIOLOG GEN^TM^ III plates.

### 2.3. Soil Physical, Physicochemical, and Chemical Analyses

Soil samples were sieved through a 2 mm mesh and air dried. Particle size distribution (clay, silt, and sand contents) was analyzed by the modified hydrometer method [53]. The pH was determined in water (1:2.5 *w*/*v*). Soil organic C (SOC) concentration was analyzed using a ground subsample of 5 g of fresh soil that passed through a 100-mesh screen in an automated C analyzer (SHIMADZU 5005A^®^, Kyoto, Japan); concentrations of total soil N (TN) and total soil phosphorus (TP) were determined from the acid digestion in H_2_SO_4_ concentrated procedure [54] using an NP elemental analyzer (Technicon Autoanalyzer III). Analysis of variance (ANOVA) and Tukey’s HSD mean comparisons (α = 0.05) were used to depict differences of the soil parameters between localities and management categories.

### 2.4. DNA Extraction and 16S rRNA Sequencing

Genomic DNA was extracted from 0.5 g of fresh soil by triplicate, using the MoBio Power Soil DNA commercial kit (MoBio Laboratories, Solana Beach, CA, USA) according to the manufacturer’s instructions. Genomic DNA samples were submitted to the Research and Testing Laboratory (Lubbock, TX, USA, https://rtlgenomics.com/, accessed on 21 August 2022) for 16S rRNA gene sequencing of the V1-V2 region (27F/388R) [55,56] using an Illumina MiSeq^TM^ instrument (Illumina, San Diego, CA, USA).

### 2.5. 16S rRNA Sequence Data Processing

Illumina raw sequences (6,097,749) were processed with QIIME 2 v 2018.8 [57]. Chimera identification and amplicon sequence variants (ASVs) clustering were performed using the DADA2 algorithm implemented in QIIME 2. Sequences were trimmed by 30 base pairs (both forward and reverse) and truncated at 220 base pairs during the ASV clustering in DADA2. The filtered sequences (2,272,116) were assigned to 16,196 ASVs. Taxonomic identity of the resulting ASVs was assigned with the Greengenes DataBase (v13.8). Due to the compositional nature of the metagenomic data [58], a center log ratio (CLR) normalization was performed with the MicrobiomeAnalyst web-based tool [59]. The raw data (paired end reads) were deposited in the NCBI sequence read archive (SRA) with the accession number PRJNA753244.

### 2.6. Carbon Mineralization Measurements

We used BIOLOG-GEN^TM^ III plates (Haywood, CS, USA) to evaluate the soil microbial communities’ C utilization as a proxy of the C_MIN_ of bacteria (Gram-positive and Gram-negative). The BIOLOG-GEN^TM^ III plates were 96-well microtiter plates containing different substrates that represent common root exudates and soil compounds [60], which can be classified in functional guilds [61]. Specifically, the 76 C sources present in the BIOLOG-GEN^TM^ III plates were analyzed in terms of six chemical families (guilds): (i) amines/amides (d-glucuronamide), (ii) amino acids (d-serine and l-alanine), (iii) carbohydrates (d-maltose, d-cellobiose, and Sucrose), (iv) carboxylic acids (l-lactic acid and d-malic acid), (v) miscellaneous (Glycerol and Pectin), and (vi) polymers (dextrin and tween40). We prepared soil suspensions for the inoculation of microplates with 3 g of soil and 45 mL of salt solution (0.85% NaCl) in sterile conical 50 mL tubes that were vortexed for 10 min. After vortexing, the soil suspensions were centrifuged at 1000 rcf. Each BIOLOG-GEN^TM^ III well was inoculated with 100 µL aliquots of the soil suspensions (0.1 OD_595 nm_). Plates were incubated for 48 h at room temperature. The intensity of color development in the BIOLOG-GEN^TM^ III plates, as an indicator of C source utilization, was quantified spectrophotometrically with the BioTek^TM^ Epoch^TM^-2 instrument (Agilent Technologies IL, Santa Clara, CA, USA). We estimated bacterial community-level changes in C utilization with matrices constructed with the average well color development per functional guild that were analyzed by ANOVA’s test and Tukey HSD mean comparisons test at *p* < 0.05.

### 2.7. Data Analyses of Microbial Diversity

Alpha diversity. We calculated Chao1 diversity index from the ASV table using the *phyloseq* R package [62]. Permutational analysis of variance (PERMANOVA) and Wilcoxon-tests were applied to evaluate differences between treatments using the *ggsignif* R package [63].

Beta diversity. Aitchison distance matrix was calculated from the ASV table and non-metric multidimensional scaling (NMDS) analysis was performed using *coda.base* and *phyloseq* R packages. Further, a PERMANOVA test and random forest (RF) analysis, using a mean decreased accuracy algorithm, were applied to test for differences across management categories, using the Microbiome Analyst web-based tool [59], at the order level because of the resolution of deeper taxonomic categories (i.e., the percentage of non-assigned ASV to the order (15.20%), family (33.80%), genus (66.38%), and species (97.88%) levels). Only the bacterial orders that were differentially abundant in terms of the mean decrease in accuracy (MDA) parameter (MDA < 0.025) in the RF analysis were represented in the NMDS. MDA is defined as “the decrease in model accuracy classification from permuting the values in each feature” [64].

### 2.8. Carbon Source Utilization Estimates

Carbon mineralization was measured with BIOLOG^TM^ GEN III plates for all soil samples. The capability of microorganisms to utilize different C sources was estimated with the average well color development (AWCD) [65,66] (Equation (3)). To evaluate differences in carbon mineralization for the various C source guilds across different management categories we followed ANOVA tests.

(3) $$AWCD=\overset{n}{\underset{I=1}{\sum }}\frac{\left(Ci-R\right)}{n}\;$$

Equation (3). Average well color development, where *C* represents the absorbance value of control wells, *R* is the absorbance of the response wells, and n is the number of *C* substrates for each C guild. Values of (*Ci − R*) less than 0.06 are calculated as zero [67].

### 2.9. Integrating Microbial Composition, Management Categories, Soil Properties, and C_MIN_

To examine the relationships between microbial diversity, management intensity, soil properties, and the utilization of different carbon sources, we followed an NMDS multivariate analysis. Specifically, we coupled in this analysis the distance matrices for (i) microbial composition (Aitchison distance) and (ii) soil properties and AWCD (Bray–Curtis distance). Furthermore, graphically, we highlighted the differentially abundant bacterial orders identified through RF analysis.

## 3. Results

### 3.1. Soil Physical, Physicochemical, and Chemical Analyses

The results of the analyses of variance for soil properties indicated significant differences across management categories but not across localities. In particular, the analysis of variance of soil properties among the three localities (Naolinco, Coatepec, and Huatusco) indicated that inter-locality variation was statistically indistinguishable (Table S2; *p* > 0.05). In contrast, we found significant differences for some soil properties depending on management category (Table 1). For instance, soil moisture and pH were lower in managed agricultural ecosystems compared to non-managed sites. In addition, total N concentration across all management categories was lower than under non-managed sites, while the lowest P concentration was found in the low-management category samples. Finally, although no significant changes in soil organic carbon (SOC) concentrations were detected with land-use change, the C:N ratio varied significantly across management categories, increasing in value with increased management.

### 3.2. Microbial Diversity

**Figure 2:**
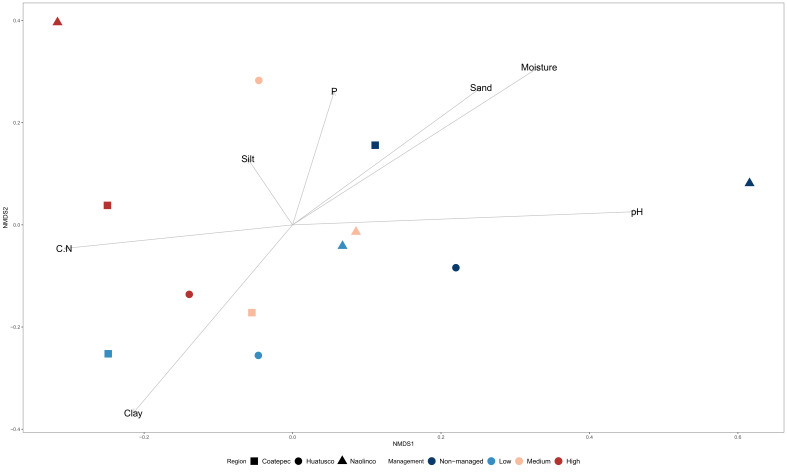
Bacterial community composition dissimilarity across management categories in coffee agroecosystems. Multidimensional scaling (MDS) ordination of microbial communities by management intensification and localities with physicochemical variables overlapped.

**Figure 3:**
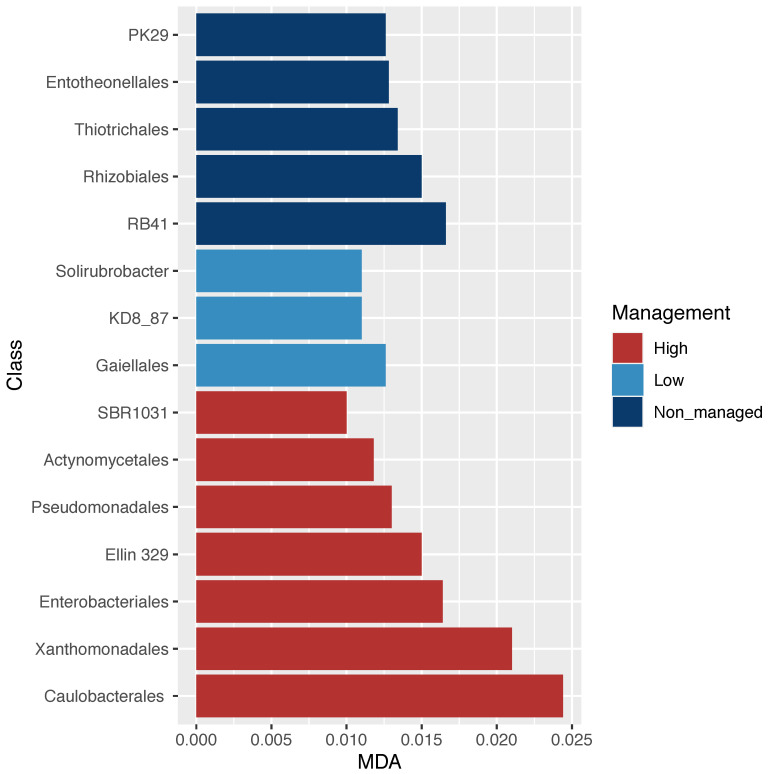
Bacterial order dissimilarity abundance across management categories. Random forest analysis; significant orders associated with management categories based on the mean deceased accuracy algorithm (MDA < 0.025). Alphanumerical orders annotated in the Greengenes database (PK29; RB41; KD8_87; SBR1031; Ellin 329), refer to new orders that have not been fully described.

The analyses of microbial community diversity (alpha diversity, Chao1 index) did not show differences across samples for localities or management categories (Figure S1). In contrast, when community composition (beta diversity) was analyzed, through non-metric multidimensional scaling, differences across samples based on management categories but not on localities were observed (Figure 2). Moreover, ordination based on community composition differences was related with differences in C:N and pH between managed and non-managed categories (Figure 2). The relative abundance of microbial orders (Figure S2) was used to identify specific bacterial orders statistically associated with management categories. Through RF analysis, specific bacterial orders were identified with three management categories (non-managed, low-management, and high-management), as shown in Figure 3.

### 3.3. Carbon Mineralization

All samples showed the capacity to utilize all C sources in the BIOLOG-GEN^TM^ III plates, indicating high diversity of C_MIN_ capabilities. Nonetheless, for the most part, no significant differences in C_MIN_ rates (AWCD) were detected across management categories (Figure S3). The only exception was for the amides/amines guild, where samples from highly managed sites showed a significantly lower utilization of this C source guild compared to non-managed category samples (Figure S3).

### 3.4. Integrating Microbial Composition, Management Categories, Soil Properties, and C_MIN_

**Figure 4:**
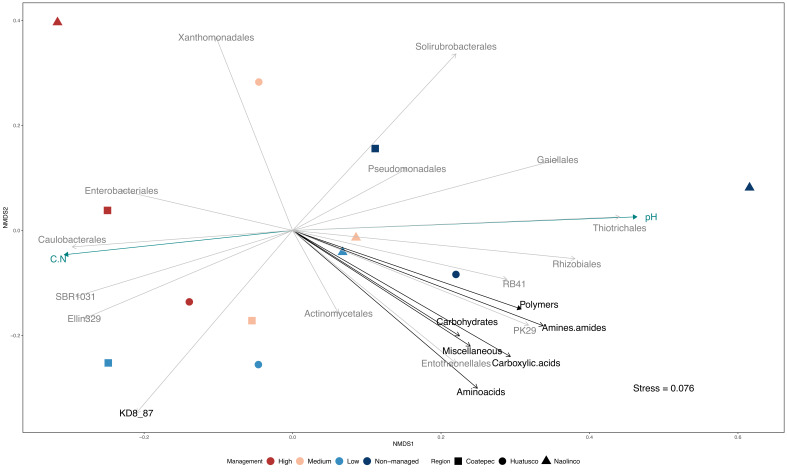
Bacterial community composition and carbon mineralization dissimilarity across management categories. Multidimensional scaling (MDS) ordination of microbial communities by management intensification and localities. Statistically significant microbial orders and physicochemical variables were overlapped.

To further investigate the relationships among management categories, soil properties, and specific bacterial groups, we followed a coupled multivariate analysis approach. The results of this approach showed a distinction among management categories, based on microbial composition (Figure 4 and Figure S2). Moreover, microbial composition differences and management categories appeared to be related to the variation in specific soil properties, such as pH and C:N, and in turn with C_MIN_ estimates, defined here as AWCD. For instance, the microbial composition of highly managed sites appeared to be associated with more acidic soils and higher C:N ratios. In addition, based on the identification of differentially abundant bacterial orders (RF analysis; Figure 3), the presence of groups such as *Enterobacteriales* in highly managed soils with acidic pH, high C:N ratios, and lower C_MIN_ estimates can be observed. In contrast, we found *Rhizobiales* as a group associated with non-managed sites, higher C_MIN_ estimates, less acidic soils, and lower C:N ratios. Finally, considering the arrow length and direction of vectors representing C_MIN_ estimates for the different C guilds, it is worth mentioning the lower values of these estimates in high-managed plots compared to non-managed categories.

## 4. Discussion

Soil processes such as C_MIN_ are multivariate and complex by nature. Different variables are involved in this process and can be characterized individually and further integrated with multivariate approaches. In this study, we followed a characterization of the individual components that are known to be associated with C_MIN_, specifically soil physicochemical and nutrient properties, the microbial composition of communities (through 16S rDNA amplicon sequencing), and a proxy of C_MIN_ based on C source degradation capacity (using BIOLOG GEN-III plates) as a direct functional outcome.

We identified differences in soil properties across management categories (Table 1; Figure 2). For instance, we found a reduction in total N in managed sites compared to non-managed sites, which is consistent with previous studies where the content of N in coffee agroecosystem soils was higher in low-management compared to high-management categories [68]. The lower N content in highly (or conventionally) managed sites may be related to the application of agrochemicals reducing the association of nitrogen-fixing bacteria with their plant hosts [10,69]. Additionally, we found a loss of total P only in the low-management category, which suggests the recovery of soil P through fertilization [38,70]. P fertilizers also can suppress the secretion of microbial enzymes involved in the mineralization of organic P in the long term [71], leading to a low P content when fertilizers are not supplied. In addition to these results, we observed a trend (not statistically significant) of organic C loss in managed sites, noted previously in other studies [72]. Moreover, the soil C:N ratio was higher in the high-management category than the low-management category and non-managed sites (Table S2), which could be due to changes in soil pH, as previously reported [13]. C accumulation is affected by changes in pH [73], and, as some types of fertilizers acidify soils (i.e., inorganic N fertilizers often supplied as NH_4_^+^), fertilization can lead to delayed microbial growth and organic matter decomposition [10,74], which, in turn, increases soil the C:N ratio [75]. In addition, the observed C:N differences across management categories may be related to the differences in both quantity and quality of C and N inputs either through natural litter deposition or fertilization practices [76,77]. Overall, and in accordance with previous studies, the observed correlations of microbial composition with soil physicochemical variables (Figure 2) could be due to conditions associated with specific management practices that affect microbially mediated processes [10].

In this study, we identified bacterial orders whose abundance was statistically related to specific management categories (Figure 3). For instance, the highest abundance of *Rhizobiales* in the non-managed sites may suggest good soil quality associated with abundant nitrogen-fixing bacteria [5]. *Rhizobiales* are typically symbiotic nitrogen-fixing bacteria that can be found in surrounding soil besides their association to a host; the major factors that determine their abundance are environmental variables such as soil acidity [69]. The lower abundance of *Rhizobiales* in the managed sites could be attributed to the removal of native vegetation and the addition of fertilizers that acidify soil, which is in accordance with previous studies on land-use conversion (i.e., the abundance of *Bradyrhizobium diazoefficiens* and *Rhizobium* sp. was negatively correlated with nitrogen fertilization (NH_4_^+^)) [78,79,80]. In contrast, highly managed sites had a greater abundance of *Enterobacteriales*, frequently associated with an anthropogenic influence [81], including the intensification of land use [82]. Moreover, the *Xanthomonadales* order is also abundant in high-management category sites and has been associated with environments contaminated with acids and nitrates [83], which in our study may be related to the use of chemical fertilizers. For instance, the use of fertilizers in long-term experiments has been shown to be related to the high abundance of both *Xanthomonadales* and *Enterobacteriales* [84]. Overall, these results show that management intensification alters bacterial composition in soil, probably due to pH change associated with fertilization.

Microbial diversity is considered to play a significant role in global C cycling, but the bacterial community structure is frequently left out or black boxed in stoichiometric nutrient cycling studies [42,85]. In contrast, in the present study, using a multivariate approach, we made explicit the role of microbial diversity in nutrient cycling through the integration of microbial composition, soil physicochemical characteristics, locality, and management categories, which allowed us to investigate how these components were related, emphasizing the effect on C_MIN_ (Figure 4). In this context, we predicted shifts in the microbial composition of soil communities with land-use change and the intensification of management practices, coupled with a reduction in their functional diversity (C source range). Our prediction was confirmed as a general trend in which functional diversity was reduced while intensification increased (Figure S3 and Figure 4) and specific environmental conditions and bacterial groups could be associated with this trend. For instance, a broader range of C source utilization (amines/amides guild) was correlated with a high abundance of specific bacterial orders such as *Actinomycetales*, *Entotheonellales*, PK29, and RB41 in low-management categories (Figure 4). On the one hand, *Actinomycetales* are commonly found in soils and are known for having a significant role as saprophytes, breaking down complex organic matter into more readily assimilable nutrients [86,87]. Moreover, *Actinomycetales* order has been recognized as a major contributor to the metabolism of carbohydrates and amino acids in different environments, including cultivated soils [88,89,90]. On the other hand, little is known about the carbon use of *Entheonellales* and PK29, although RB41 has been reported as including taxa influencing carbon balance in different environments [91]. Our integrated approach allowed us to appreciate the specific relationship between bacterial orders and the microbially mediated processes of carbon mineralization while looking at the influence of different management strategies in both.

Technical and analytical developments in environmental microbiology, such as those associated with approaches that are not limited by culture and the possibility of studying entire communities instead of populations, have proven to further our understanding of the ecological role of microorganisms. However, the limitations of these approaches should be acknowledged and reflected upon. For instance, when dealing with specific genomic regions through amplicon sequencing, several biases are introduced associated with the specific region to be sequenced and the PCR amplification process [92,93]. One of the consequences of these biases is the limited taxonomic assignation at lower levels, as databases are filled with sequences of uncharacterized and non-cultivated groups. Furthermore, there are limitations in the assignment of the functional role of different groups based solely on 16S rDNA partial sequences [94,95], hindering our understanding of the mechanisms responsible for the observed transformations. Nonetheless, the coupling of multiple approaches and the use of multivariate statistics can help to partially overcome these limitations while looking at the patterns at the system and community levels and identifying trends upon which specific hypotheses to be further tested can be constructed. In our case, we identified that *Caulobacterales*, *Enterobacterales,* and *Xanthomonadales* may be responsible for the reduced range of C source utilization and high C:N ratio in cases of high-intensity management. This corresponds to a specific hypothesis that can be tested experimentally focusing on the specific groups through culture enrichment strategies [96] and tracking their C utilization of specific substrates using qPCR or SIP techniques [97,98].

## 5. Conclusions

We have shown that land-use change and microbial composition are correlated, and specific microbial orders were identified as linked to a broader utilization range of carbon sources. The identification of specific bacterial groups, management practices, and soil parameters, and their correlation with carbon source degradation capacity (C_MIN_), offers the possibility to generate hypotheses to be further tested on the interplay of all these components, either in the field or in the laboratory, and to further our understanding of agroecosystem functioning and sustainable management.

## Figures and Tables

**Table 1:** Soil properties of the four studied management categories (non-managed, low, medium, high).

Variable	Management Category				
	Non-Managed	Low	Medium	High	F-Value; *p*-Value
Clay (%)	34.0 a ± 1.16	50.7 a ± 4.67	37.3 a ± 4.67	47.3 a ± 3.71	3.746; 0.080
Silt (%)	18.7 a ± 0.67	21.3 a ± 6.36	29.3 a ± 1.76	24.7 a ± 1.76	1.796; 0.226
Sand (%)	47.3 a ± 1.76	28.0 b ± 3.06	33.3 ab ± 3.53	28.0 b ± 5.03	6.640; 0.015
Soil moisture (%)	4.39 a ± 0.14	3.25 b ± 0.09	3.45 b ± 0.20	3.38 b ± 0.08	15.93; <0.001
Total N (mg g^−1^)	8.32 a ± 0.9	4.73 b ± 0.37	5.77 b ± 0.4	4.36 b ± 0.18	14.76; <0.001
Total P (µg g^−1^)	272 a ± 34	122 b ± 15	173 ab ± 48	184 ab ± 43	3.287; 0.037
SOC (mg g^−1^)	27.6 a ± 3.55	19.1 b ± 1.53	22.8 ab ± 1.63	20.9 ab ± 0.65	2.816; 0.057
C: N	3.3 b ± 0.19	4.0 ab ± 0.19	4.0 ab ± 0.25	4.8 a ± 0.3	7.858; <0.001
pH (H_2_O)	5.3 a ± 0.27	4.3 b ± 0.27	4.6 b ± 0.1	4.0 b ± 0.15	10.86; <0.001

Values are means with standard error between parentheses. Different letters indicate that means are significantly different (*p* < 0.05) between management categories.

## Data Availability

16S rDNA sequence raw data (paired end reads) were deposited in the NCBI sequence read archive (SRA) with the accession number PRJNA753244.

## Supplementary Materials

The following supporting information can be downloaded at: https://www.mdpi.com/article/10.3390/microorganisms10091763/s1. Figure S1: Soil microbial community diversity of coffee agroecosystems and non-managed plots. Figure S2: Order-level taxonomic composition of soil microbial communities for different management categories of coffee agroecosystems and non-managed plots. Figure S3: Carbon mineralization diversity for coffee agroecosystems and non-managed soil samples. Table S1: Index management for studied coffee agroecosystems. Table S2: Soil properties of the three studied localities (Naolinco, Coatepec and Huatusco. Values are means with standard error between parentheses. Non-significant differences among region samples were found (ANOVA test).
