# Supplementary material for: Land-Use Change and Management Intensification Is Associated with Shifts in Composition of Soil Microbial Communities and Their Functional Diversity in Coffee Agroecosystems
Source: Microorganisms. 2022 Aug 31;10(9):1763. doi: 10.3390/microorganisms10091763 (PMC9504970; doi:10.3390/microorganisms10091763)
Supplement: Supplementary file 1 [file microorganisms-10-01763-s001.zip › microorganisms-1815566-supplementary.pdf]

Supplementary

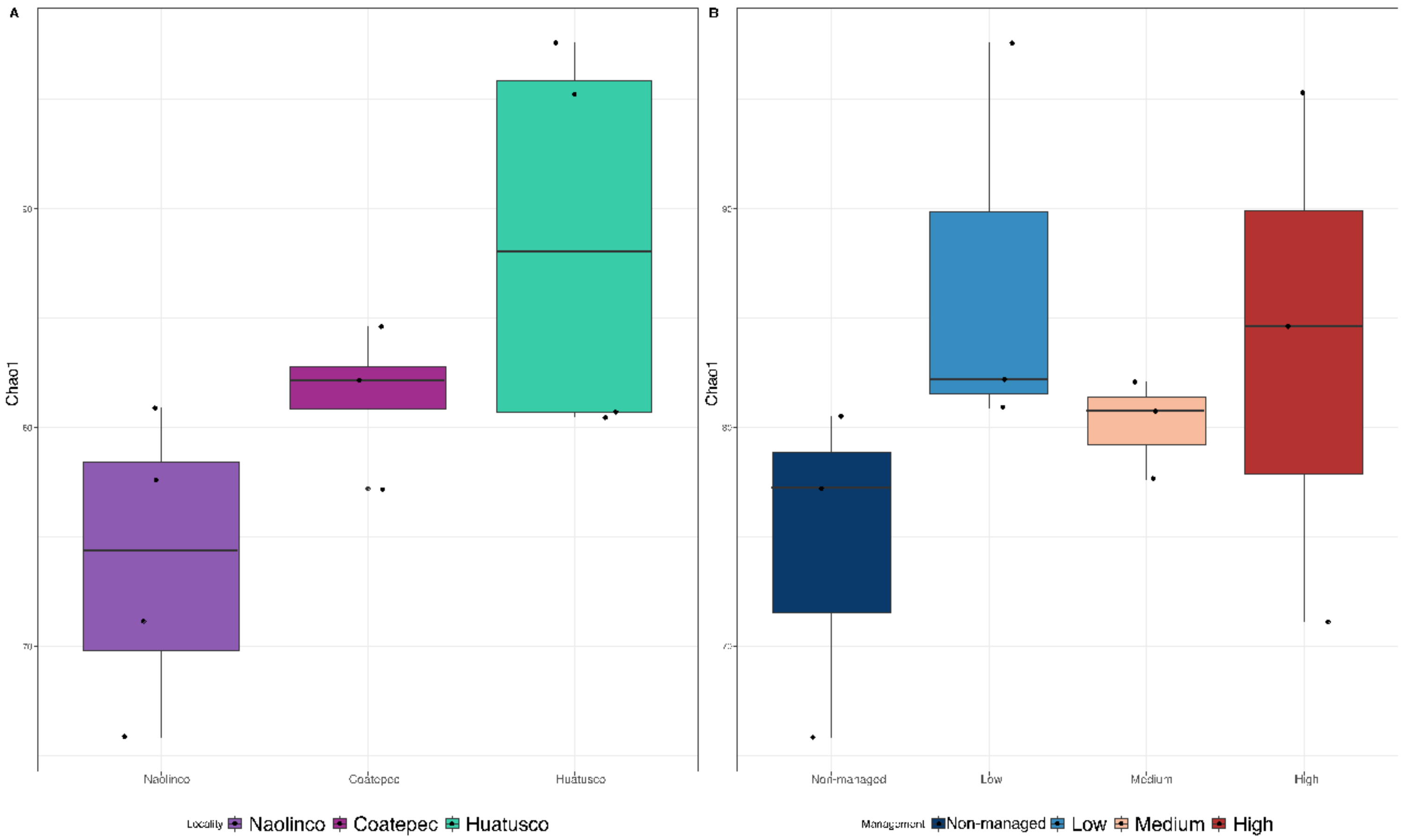

Figure S1. Soil microbial community diversity of coffee agroecosystems and non-managed plots. Chao 1 index diversity across localities (A) and management (B) categories.

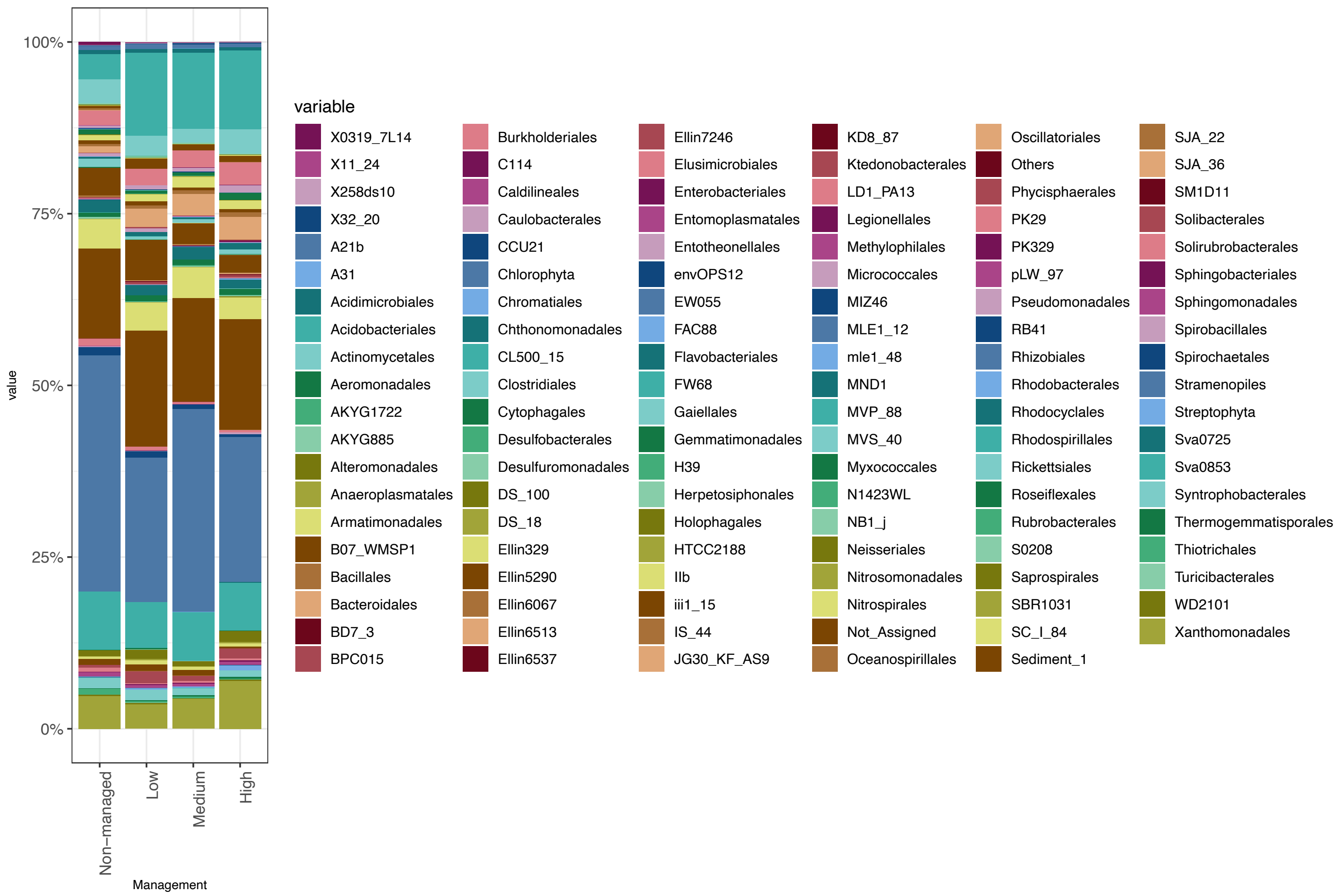

Figure S2. Order-level taxonomic composition of soil microbial communities for different management categories of coffee agroecosystems and non-managed plots. Each bar represents the average relative abundance of each bacterial taxon within a group at order level. Alpha-numerical order names correspond to Greengenes DataBase (v13.8) assignment.

Table S1. Index management for studied coffee agroecosystems. [A] Values obtained through the survey applied to coffee producers: none (0), little to medium (0.5) and a lot (1.0) level of use of each variable. [B] Mean of management variables use as index management. [C] Management category was defined into three categories according to the 0-0.5 range of the Index registered: Low (0-0.167), Medium (0.167-0.334), and High (0.334-0.501).

Site	Management variables [A]							Index [B]	Management category [C]	Locality
	Tractor	Yoke	Irrigation	Chemical fertilizer	Pesticides	Herbicides	Fungicides			
**CVO1**	NA							0	Non-managed	Naolinco
**CV2**	0	0	0	0	0	0	0	0	Low	
**CV3**	0	0	0	0.5	0	0.5	0.5	0.21	Medium	
**CV1**	0	0	0.5	0.5	0.5	1	0.5	0.43	High	
**CVO2**	NA							0	Non-managed	Coatepec
**CV4**	0	0.5	0.5	0	0	0	0	0.14	Low	
**CV6**	0	0	0	0.5	0	0	1	0.21	Medium	
**CV5**	0.5	0	0.5	0.5	0.5	1	0.5	0.5	High	
**CVO3**	NA								Non-managed	Huatusco
**CV10**	0	0	0	0.5	0	0	0.5	0.14	Low	
**CV12**	0	0	0	0	0.5	1	0.5	0.29	Medium	
**CV11**	0	0	0	1	0.5	1	1	0.5	High	

Table S2. Soil properties of the three studied localities (Naolinco, Coatepec and Huatusco. Values are means with standard error between parentheses. Non-significant differences among region samples were found (ANOVA test).

**Variable**	**Locality**		
	**Naolinco**	**Coatepec**	**Huatusco**
Clay (%)	38.00 ± 6.73	47.00 ± 8.71	47.50 ± 12.04
Silt (%)	25.00 ± 6.83	22.50 ± 7.72	23.00 ± 6.83
Sand (%)	35.50 ± 10.11	33.50 ± 12.04	33.50 ± 9.98
Soil moisture (%)	3.80 ± 0.66	3.40 ± 0.72	3.62 ± 0.51
Total N (mg g^{-1})	0.7 ± 0.27	0.54 ± 0.22	0.50 ± 0.10
Total P ($\mu g\ g^{-1}$)	275 ± 91	150 ± 50	200 ± 20
SOC (mg g^{-1})	43.28 ± 15.81	34.07 ± 9.25	37.05 ± 8.90
C: N	6.29 ± 0.8	6.58 ± 0.19	7.38 ± 0.97
pH (H_2O)	4.96 ± 1.09	4.075 ± 0.34	5.065 ± 0.81

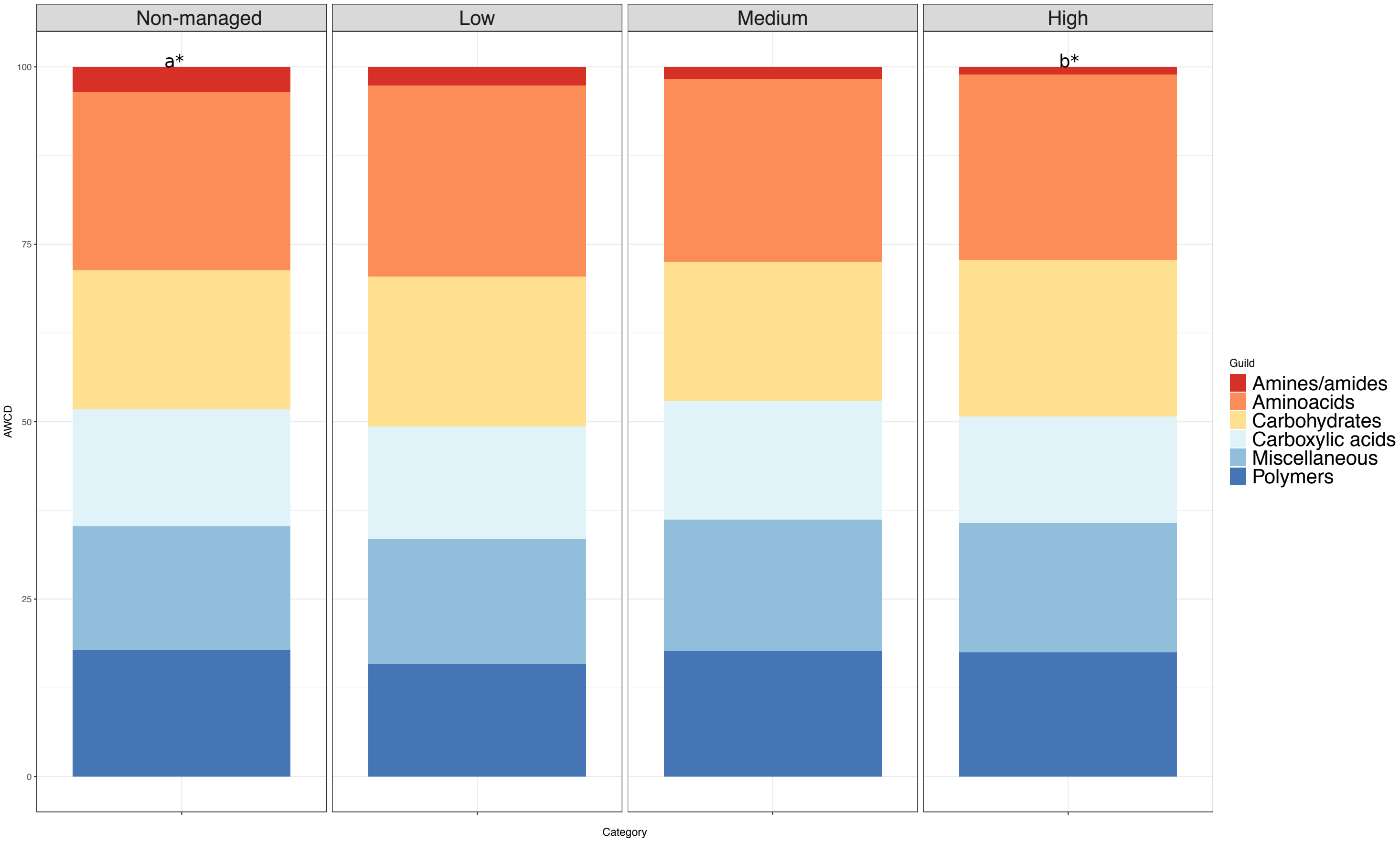

Figure S3. Carbon mineralization diversity for coffee agroecosystems and non-managed soil samples. Relative Average Well Colour Development (AWCD) tested in BIOLOG GN™ III plates by C guild. *Significant difference between management category samples (ANOVA and Tukey HSD). Different letters indicate that means are significantly different among samples.
